# 造血干细胞移植后侵袭性真菌病中国专家共识（2023年版）

**DOI:** 10.3760/cma.j.issn.0253-2727.2023.02.002

**Published:** 2023-02

**Authors:** 中国医药教育学会真菌病专业委员会 中华医学会血液学分会

造血干细胞移植（HSCT）是治疗血液系统疾病的有效手段。侵袭性真菌病（invasive fungal disease, IFD）是HSCT后的重要并发症。近年来IFD诊断方法的改进，抗真菌药物种类的进一步丰富，真菌药敏试验及抗真菌药物浓度监测的使用，进一步优化了IFD患者的诊治策略。基于中国HSCT患者IFD的流行病学数据、HSCT人群特点、中国专家IFD诊治经验以及IFD诊治进展[1]–[7]，中国医药教育学会真菌病专业委员会联合中华医学会血液学分会组织国内相关领域专家制定了本共识，旨在为HSCT患者IFD的防治提供合理临床路径。

一、HSCT患者IFD流行病学特征

（一）HSCT患者IFD的发生率和死亡率

由于移植后免疫重建耗时较长以及免疫抑制药物的使用，HSCT患者IFD的发生率较高。IFD（确诊/临床诊断）多发生于HSCT后6个月内，发生率为3.4％～7.7％，在异基因造血干细胞移植（allo-HSCT）中的发生率为7.8％～13.1％，在自体造血干细胞移植（auto-HSCT）中的发生率为1.2％～4.0％[8]–[13]。确诊/临床诊断IFD的HSCT患者移植后1年死亡率为18.0％～65.3％，显著高于未发生IFD的患者；allo-HSCT后并发IFD患者的1年死亡率达36.0％～72.0％[8]–[11]。

（二）HSCT患者IFD的常见病原体分布

HSCT后IFD最常见的病原体是曲霉（*Aspergillus*），在所有IFD患者中占50.7％～88.0％；其次是念珠菌（*Candida*），占27.5％～39.0％；另外，HSCT后IFD的病原体还包括毛霉目真菌（*Mucorales*）、镰刀霉（*Fusarium*）、尖端赛多孢菌（*Scedosporium apiospermum*）、耶氏肺孢子菌（*Pneumocystis Jirovecii*）和隐球菌（*Cryptococcus*）等[8],[10],[12],[14]。

（三）HSCT患者发生IFD的危险因素

HSCT患者发生IFD的危险因素包括：①患者因素：高龄、原发病活动期、移植前IFD病史、合并糖尿病；②供者因素：人类白细胞抗原（HLA）不合、遗传易感性（如供/患者TLR4、PTX3、Dectin-1基因多态性）[15]；③移植相关因素：粒细胞缺乏、重度急性移植物抗宿主病（GVHD）、慢性GVHD、巨细胞病毒感染、糖皮质激素的使用等[8]–[9],[11]–[12]。

二、HSCT患者IFD的预防性治疗

（一）初级预防性治疗

初级预防性治疗是指具有IFD高危因素患者出现感染症状前预先应用抗真菌药物防止IFD发生[16]。预防性治疗能显著降低IFD发生率[9],[17]–[18]。可选择药物包括三唑类、棘白菌素类和两性霉素B。

推荐：拟行allo-HSCT的患者接受抗IFD初级预防，预防治疗与移植前预处理同时开始，至少持续至移植后3个月，合并急性或慢性GVHD或接受免疫抑制治疗时，疗程应延长至GVHD临床症状控制且免疫抑制剂减停为止。

推荐：以氟康唑作为真菌感染低风险患者（IFD发生率低于5％）植入前预防用药；以泊沙康唑、伏立康唑作为IFD发生风险较高人群的植入前预防用药[19]–[21]；将棘白菌素类作为三唑类药物禁忌证患者的预防性抗真菌用药[22]–[24]。

推荐：植入后，allo-HSCT合并急性或慢性GVHD及接受免疫抑制药物治疗时继续使用抗真菌药物以预防IFD。泊沙康唑可作为allo-HSCT植入后合并急性或慢性GVHD患者的IFD初级预防治疗药物[25]。泊沙康唑在预防GVHD患者侵袭性曲霉病和降低与IFD相关的死亡率较氟康唑具有显著优势，伏立康唑可作为植入后GVHD患者IFD初级预防的备选方案[20]–[21]。棘白菌素类可作为患者IFD预防的选择之一。

（二）再次预防性治疗

再次预防性治疗是指对于既往有确诊或临床诊断IFD病史的患者，在IFD达到完全或部分缓解后接受HSCT时给予抗真菌药物以防止IFD再次发生。推荐：使用有效抗IFD治疗药物用于allo-HSCT患者再次预防，至少持续至移植后3个月。若患者合并GVHD接受免疫抑制药物治疗，则疗程应延长至GVHD临床症状控制且免疫抑制剂减停为止[26]。

IFD病史曾被视为HSCT禁忌证。随着IFD早期诊断、新型治疗药物等进展，IFD病史不再是HSCT的绝对禁忌证。但由于既往IFD病史可使HSCT后IFD的复发风险及HSCT后死亡率显著增加，在HSCT前合理评估适宜患者及HSCT时机具有重要意义，但目前还缺乏相应的评估体系。评估参数应包括患者原发病的状态及治疗计划、IFD的疗效、HSCT相关风险、实施再次预防性治疗策略的可行性及有效性等。

对肺部霉菌病（IMD）患者，在患者治疗4周以上并证实疗效的基础上，接受HSCT是可行的。但应综合评估原发病的风险及IMD复发的风险。部分患者可在HSCT前实施手术治疗，适用于非播散性IMD、血液病缓解或稳定状态、体能状态及共存病可耐受手术、经充分治疗仍持续存在的孤立性病灶、有间断咯血等症状。念珠菌病（包括播散性念珠菌病）在有效治疗及合理再次预防情况下，复发风险极低，不应作为HSCT禁忌证。隐球菌病经过充分治疗，血清隐球菌抗原转阴或显著下降后，再次预防性治疗情况下复发风险较低，HSCT相对安全。

建议：具有IFD病史患者，对移植适应证及移植时机进行个性化评估。建议至少治疗4周以上，并证实IFD获得改善（包括临床症状、影像学、生物标记）。如原发病许可，尽可能在移植前争取更长的抗真菌治疗时间。

三、预防性治疗后突破性IFD的处理

（一）定义

突破性IFD定义为在应用抗真菌药物治疗期间，初级或再次预防7 d后至预防性治疗停止7 d内发生的任何IFD，包括抗真菌药活性范围之外真菌感染[27]–[28]。

（二）发生率和致病菌分布

allo-HSCT患者接受真菌初级或再次预防后突破性IFD的发生率为0.4％～3.6％[16],[28]。主要致病菌为曲霉，其次为念珠菌、毛霉等。根据预防性抗真菌药的种类、当地真菌流行病特征以及患者特征的不同，发生率及致病菌分布有一定差异。

（三）诊断流程

预防治疗后尤其是广谱抗真菌药预防后突破性IFD诊疗流程需结合患者IFD危险因素、预防用药、IFD突破临床特征等进行处理[28]。

建议：寻找突破性IFD发生的可能原因，除外因预防药物无效的真菌病原体二次感染、耐药真菌的感染、中心静脉置管的定植菌感染、预防药物未达到有效浓度等原因[27]。

建议：在治疗性药物浓度检测（TDM）可及的情况下对患者体内唑类药物血药浓度进行监测。

建议：积极进行病原学诊断，包括培养、镜检等；广谱抗真菌药物预防（覆盖曲霉）条件下半乳甘露聚糖（GM）试验敏感性和阴性预测价值降低，此时肺泡灌洗液GM试验仍具有临床意义。建议原则上可行支气管镜检查（48～72 h内）和（或）CT引导下的病变组织活检以推动特异性诊断。PCR、微生物宏基因组二代测序（mNGS）可为诊断提供一定参考信息。

（四）治疗原则

积极寻找突破性IFD的原因和诊断流程是正确治疗的基石。建议：因药物浓度不足所致，提高至有效浓度；排除药物浓度不足因素的患者，建议更换抗真菌药种类；对明确菌种的患者，可参考体外药敏试验选择抗真菌药物种类。

建议：综合考量感染进展速度、严重程度以及本地IFD流行病学，应用个体化治疗。病情危重的患者应尽快启动治疗。初始治疗时，依据患者预防用药种类、突破性IFD的可能致病菌选择广谱、强效的抗真菌治疗方案，且尽可能选择与预防药物不同类别的抗真菌药物。积极获得病原学结果，根据真菌检测及药敏结果，综合选择治疗药物；为避免交叉耐药的存在，也应尽量选择不同类别的抗真菌药物。

抗真菌药物治疗基础上，辅助手段包括减少潜在的免疫抑制药物用量、G-CSF应用、手术治疗。

四、HSCT患者IFD的目标治疗

IFD目标治疗是指患者达到临床诊断或确诊IFD标准而进行抗真菌治疗。感染病原菌较明确，可依据真菌种类、药物抗菌谱及患者具体病情选择用药。可选择的药物包括三唑类、棘白菌素类、两性霉素B。

（一）侵袭性曲霉病

1. 侵袭性肺曲霉病（IPA）：随机对照试验表明，相比于其他抗真菌药物，伏立康唑对于侵袭性曲霉病反应率更高，可显著降低死亡率；另有三期临床试验表明，艾沙康唑与伏立康唑疗效相似但安全性和耐受性可能更佳[29]–[30]。推荐：伏立康唑、艾沙康唑、泊沙康唑作为确诊/临床诊断侵袭性曲霉病的一线用药[31]。建议：以两性霉素B、棘白菌素类作为有三唑类药物禁忌证患者的替代用药[32]，具体疗程根据临床感染严重程度、相关症状和体征恢复速度、影像学结果、GVHD、免疫抑制状态等情况决定[1]。对于单药治疗失败或无法耐受、多部位感染或耐药真菌感染的高危病例，建议采用两种药物联合治疗。机制不同的抗真菌药物联合可能对高危侵袭性曲霉病更有效，如棘白菌素类药物联合伏立康唑或脂质体两性霉素可能进一步提高治疗反应，对临床诊断IPA患者有可能提高生存率[33]–[34]。

对于以下患者可实施手术治疗：非播散性IPA、血液病缓解或稳定状态、体能状态及共存病可耐受手术、经充分治疗仍持续存在的孤立性病灶、有间断咯血等症状。

2. 肺外侵袭性曲霉病：肺外常见部位主要包括中枢神经系统及鼻窦、骨、心脏（心内膜炎、心包炎、心肌炎）、眼睛（眼内炎、角膜炎）、皮肤及软组织等。

中枢神经系统曲霉感染的死亡率极高，推荐患者转诊神经外科治疗（开颅脓肿切除术、脓肿引流术、脑室分流术等）[35]–[36]。同时，与其他抗真菌药物相比，伏立康唑更容易透过血脑屏障，对中枢神经系统曲霉感染的反应率达35％[35]，推荐将伏立康唑、艾沙康唑作为allo-HSCT后中枢神经系统曲霉感染的首选药物治疗方案。建议有三唑类药物禁忌证的患者选用两性霉素B治疗[37]。

对于鼻窦曲霉感染的患者，推荐转诊耳鼻喉科治疗（切开引流术、脓肿引流术等）及使用局部抗真菌药治疗[31]。根据既往临床证据，推荐将伏立康唑和两性霉素B作为鼻窦曲霉病的药物治疗方案[30],[32]。

（二）侵袭性念珠菌病

推荐：棘白菌素类作为侵袭性念珠菌病的首选药物[38]–[39]。非危重症和无唑类暴露患者也可选择唑类[6]。患者不能耐受或无法获得上述抗真菌药物或耐药者，也可使用两性霉素B[40]–[41]。如病情稳定且血培养转阴患者、为敏感菌株感染，可选择三唑类进行降阶梯治疗。若有明确的真菌种属鉴定结果，应以药敏试验指导治疗[6]。

念珠菌血症不伴有粒细胞缺乏患者中，若初始治疗病情稳定、血培养转阴5～7 d后（初始治疗10 d以上），可改用静脉或口服唑类药物治疗，危重症等免疫力极度低下患者，初始治疗疗程相应延长。念珠菌血症合并粒细胞缺乏、且没有播散性病灶患者，应血培养转阴后至少再治疗2周，且感染征象消失、粒细胞缺乏恢复。

建议对确诊侵袭性念珠菌病患者尽早拔除中心静脉导管[38],[42]。若因病情无法及时拔管，应考虑应用棘白菌素或脂质体两性霉素B，因其对念珠菌生物膜治疗活性较好[43]–[44]。

（三）耶氏肺孢子菌肺炎治疗

推荐：①复方磺胺甲噁唑（SMZ）/甲氧苄啶（TMP）；②氨苯砜联合TMP；③克林霉素联合伯氨喹；④阿托伐琨。疗程推荐21 d。

对于危重患者，可根据病情选择联用糖皮质激素。

（四）毛霉病治疗

推荐：在移植患者体能及病情可耐受的情况下尽量手术治疗。

毛霉病的一线药物为两性霉素B，二线药物包括泊沙康唑和艾沙康唑。

五、HSCT后抗真菌药物的监测管理

（一）抗真菌药物的TDM

抗真菌领域中，TDM通常用于具有非线性药代动力学性质且药物相互作用较多的三唑类药物。临床极少出现需对氟康唑行TDM的情况（例如行血液透析的重病患者），故对氟康唑行TDM的推荐缺乏足够证据。此外，目前尚缺乏证据支持对多烯类或棘白菌素类药物行TDM。适用TDM的三唑类药物包括伏立康唑、泊沙康唑、伊曲康唑等，此类药物治疗窗窄、个体差异大，且血药浓度与疗效和毒性均有相关性，因此推荐在预防或治疗失败时可行TDM寻找是否存在药物浓度不足，在出现器官损伤时可行TDM以明确药物浓度是否过高。

（二）合理应用TDM管理抗真菌药物和免疫抑制剂的相互作用

三唑类抗真菌药（泊沙康唑、伏立康唑、氟康唑、伊曲康唑等）在体内代谢主要以其环上的氮原子与细胞色素P450（cytochrome P450, CYP）酶系统的血红素铁结合，从而抑制肝内CYP酶系活性，其中以CYP3A4为主要抑制对象。CYP3A4既是三唑类抗真菌药物的共同代谢酶，也是免疫抑制剂代谢的重要代谢酶，这使得三唑类药物在HSCT患者中应用时需考量与免疫抑制剂间的药物相互作用问题。棘白菌素类药物如卡泊芬净、米卡芬净等与P450系统中的任意代谢酶关联均较小，但其血药浓度受环孢素A影响，并对他克莫司的血药浓度有潜在影响。抗真菌药物相关肝代谢酶及抗真菌药物与免疫抑制剂相互作用见表1。

**表1:** 抗真菌药物相关肝代谢酶及免疫抑制剂相互作用

抗真菌药	P450酶代谢相关性	与免疫抑制剂相互作用
泊沙康唑	CYP3A4（强效）	与他克莫司合用可增加泊沙康唑的峰值浓度（Cmax）和药时曲线下面积（AUC）；与环孢素A合用可提升环孢素A的血药浓度；禁止与西罗莫司合用；禁止与匹莫齐特、奎尼丁合用
伏立康唑	CYP2C9，CYP3A4，CYP2C19	与环孢素A合用可增敏伏立康唑，并提升环孢素A血药浓度
氟康唑	CYP2C9，CYP3A4，CYP2C19	与环孢素A合用可明显增高环孢素A的血药浓度
伊曲康唑	CYP3A4，CYP3A5，P-gp	与环孢素A合用可增强伊曲康唑效果；与他克莫司合用可提升他克莫司浓度；环孢素A与他克莫司的应用是否潜在提升伊曲康唑的浓度尚有争议
艾沙康唑	CYP3A4	与他克莫司合用时无需减量他克莫司，但需对他克莫司行TDM以指导其剂量调整
卡泊芬净	几乎不通过P450酶系代谢	与环孢素A合用，卡泊芬净的AUC增加约35%；与他克莫司合用，可使他克莫司12 h血药浓度下降26%，但卡泊芬净浓度不受他克莫司的影响
